# Comparison of the efficiency of anti-VEGF drugs intravitreal injections treatment with or without retinal laser photocoagulation for macular edema secondary to retinal vein occlusion: A systematic review and meta-analysis

**DOI:** 10.3389/fphar.2022.948852

**Published:** 2022-07-22

**Authors:** Weijie Zou, Yuanyuan Du, Xiaoyan Ji, Ji Zhang, Hongping Ding, Jingqiao Chen, Tao Wang, Fangfang Ji, Jiang Huang

**Affiliations:** ^1^ Department of Ophthalmology, Second Affiliated Hospital of Soochow University, Suzhou, China; ^2^ Department of Ophthalmology, Changshu No.1 People’s Hospital, Suzhou, China; ^3^ State Key Laboratory of Radiation Medicine and Protection, Soochow University, Suzhou, China

**Keywords:** anti-VEGF, laser photocoagulation, macular edema, retina vein occlusion, retinal non-perfused areas

## Abstract

**Objective:** To compare the efficiency of anti-VEGF drugs intravitreal injections(IVI) treatment with or without retinal laser photocoagulation(LPC) for macular edema(ME) secondary to retinal vein occlusion(RVO).

**Methods:** The randomized controlled trials and retrospective studies including anti-VEGF drug IVI combined with retinal LPC and single IVI in the treatment of macular edema secondary to RVO were collected in PubMed, Medline, Embase, Cochrane Library, and Web of Science. We extracted the main outcome indicators including the best corrected visual acuity (BCVA), central macular thickness(CMT), the number of injections and the progress of retinal non-perfusion areas(NPAs) for systematic evaluation, to observe whether IVI + LPC could be more effective on the prognosis of RVO. We use Review Manager 5.4 statistical software to analyze the data

**Results:** 527 articles were initially retrieved. We included 20 studies, with a total of 1387 patients who were divided into the combination(IVI + LPC) treatment group and the single IVI group. All the patients completed the ocular examination including BCVA, slit-lamp test, fundus examination and Optical Coherence Tomography(OCT) test before and after each treatment. There was no statistical difference between the combination treatment group and single IVI group on BCVA(*WMD* = 0.12,95%*CI =* -3.54–3.78,*p* = 0.95),CMT(*WMD* = -4.40,95%*CI =* -21.33–12.53,*p* = 0.61) and NPAs(*WMD* = 0.01,95%*CI =* -0.28–0.30,*p* = 0.94).However, the number of IVI was decreased significantly in the combination treatment group in BRVO patients, compared to that in the single IVI group(*WMD* = -0.69,95%*CI =* -1.18∼-0.21,*p* = 0.005).

**Conclusion:** In the treatment of RVO patients with macular edema, the combination of IVI and retinal LPC neither improves BCVA nor reduces CMT significantly compared with the single IVI treatment. However, the combination treatment can decrease the number of intravitreal injections in patients with BRVO, while it is not observed in CRVO patients.

## Introduction

Retinal vein occlusion (RVO) is one of the most common retinal vascular diseases, with a global prevalence of 1–2% among people over 40 years old. According to the location of the occlusion, RVO can be divided into central retinal vein occlusion (CRVO) and branch retinal vein occlusion (BRVO), and the prevalence of BRVO is 4 times that of CRVO(Rogers et al., 2010; Khayat et al., 2018). It is generally thought that RVO may be caused by mechanical damage and local inflammation of the vascular wall, which can result in thrombosis that blocks the main vein in the retinal circulation, and leads to increasing intravascular pressure, eventually causing hemorrhage and retinal edema(Jaulim et al., 2013; Noma et al., 2019). The common complications of RVO are macular edema, neovascularization, neovascular glaucoma, vitreous hemorrhage and so on(Jaulim et al., 2013; Schmidt-Erfurth et al., 2019). Macular edema plays the most important role in the irreversible vision loss of RVO patients. Therefore, treatments for macular edema of RVO are vital to prevent the decline of visual acuity and protect visual function. In the past, retinal LPC had always been the main treatment for RVO patients before anti-VEGF drugs were applied because of its convenience, good repeatability and low costs. However, limitations to LPC treatment are also prominent. For example, it cannot improve the visual acuity or regress the macular edema significantly.

In recent years, IVI of anti-VEGF drugs has been recommended as a first-line clinical treatment because of its excellent therapeutic effect on ME in RVO patients(Holz et al., 2013; Jaulim et al., 2013; Noma et al., 2019; Schmidt-Erfurth et al., 2019). It can improve the visual acuity and decrease ME significantly, while it needs repeated injections, with the increasing medical costs and potential risks (such as infection and cardiovascular accidents, etc.).In addition, some studies(Donati et al., 2012; Farese et al., 2014; Rehak et al., 2014; Tomomatsu et al., 2016; Goel et al., 2019; Terashima et al., 2019; An and Jeong, 2020; Nourinia et al., 2020) illustrated that retinal LPC combined with IVI treatment may be more effective and could reduce the number of injections and the medical costs. However, this conclusion is still controversial. The purpose of this study is to systematically analyze 20 related studies through meta-analysis, in order to evaluate the value of combination therapy for RVO patients with ME and to provide some valuable suggestions to choose a better treatment.

## Materials and methods

This meta-analysis was performed based on the Preferred Reporting Items for Systematic Reviews and Meta-analysis guidelines.

### Search strategy

Publications were searched on PubMed, Medline, Embase, Cochrane library and Web of science until February 2022. The detailed search terms were as follows:

1) “Retinal vein occlusion” [Mesh]/[Title/Abstract]OR “Central retinal vein occlusion” [Mesh]/[Title/Abstract]OR “CRVO” [Title/Abstract]OR “Branch retinal/vein occlusion” [Mesh]/[Title/Abstract]OR “BRVO” [Title/Abstract]
2) “Macular edema” [Mesh]/[Title/Abstract]OR “Macular oedema” [Mesh]/[Title/Abstract]
3) “Vascular Endothelium growth factor” [Mesh]/[Title/Abstract] OR “VEGF” [Title/Abstract] OR “anti-VEGF” OR “anti-VEGF” [Title/Abstract] OR “lucentis” [Title/Abstract] OR “bevacizumab” [Title/Abstract] OR “ranibizumab” [Title/Abstract] OR “aflibercept” [Title/Abstract]
4) “Retinal laser photocoagulation” [Mesh]/[Title/Abstract] OR “Retinal photocoagulation” [Mesh]/[Title/Abstract]
5) Combine one and two and three and four

### Inclusion and exclusion criteria

The following inclusion criteria were used for this study:(1)research subjects: clinically diagnosed patients with ME secondary to RVO; (2)interventions: intravitreal anti-VEGF drugs injections combined with laser photocoagulation(IVI + LPC) and single intravitreal anti-VEGF drugs injections(IVI); (3)research types: randomized controlled trials(RCTs) or retrospective studies; (4)result evaluation: best corrected visual acuity(BCVA), central macular thickness(CMT), the number of injections and retinal non-perfusion(NPAs); (5)the follow-up period of the study should be more than 6 months.

The followings are the exclusion criteria for this study:(1) case reports or review articles; (2) duplicate publication; (3) research lacking sufficient information; (4) recurrent patients with ME secondary to RVO; (5) patients with other ocular diseases, such as diabetic retinopathy, glaucoma, age-related macular degeneration, hyperopia and uveitis.

### Data extraction and risk of bias assessment

The following information was collected from all the included studies: name of the first author, location, type of RVO, age, gender, intervention, BCVA, CMT, and follow-up periods. In our included studies, some authors used EDTRS letters while the others used LogMAR to represent BCVA. Therefore, we converted LogMAR into EDTRS letters(N) and make forest plots to illustrate the question for a better comparison. The conversion relationship is as follows:

$$N=100-\frac{-0.3+\log\mathrm{MAR}}{-0.02}$$

We calculated the Jadad score to evaluate the risk of bias: 1–3 scores was considered low-quality research, 4–7 scores was considered medium-quality research and 8–11 scores was considered high-quality research.

The bias including selection bias, performance bias, detection bias, attrition bias, reporting bias, and other factors was examined.

### Statistical analysis

The data analyses were performed by Review Manager 5.4 software, and the continuous variables are expressed by weighted mean difference(WMD) and 95% confidence interval(CI). The heterogeneity test was carried out by *χ* ^*2*^ test to calculate the heterogeneous index(*I* ^*2*^). We used the fixed effect model for data analysis if no statistical heterogeneity was observed between the studies(*p* > 0.10, *I* ^*2*^ < 50%).On the contrary, if statistical heterogeneity was observed, the random effect model would be used. The statistical results of the amalgamation effect are expressed by the Z value, according to which we can get the corresponding *p* value. The metrological data (BCVA, CMT and NPAs) and counting data(injection numbers) are expressed as mean ± standard deviation(SD), and the results will be illustrated by forest plots.

## Results

### Search results

**FIGURE 1:**
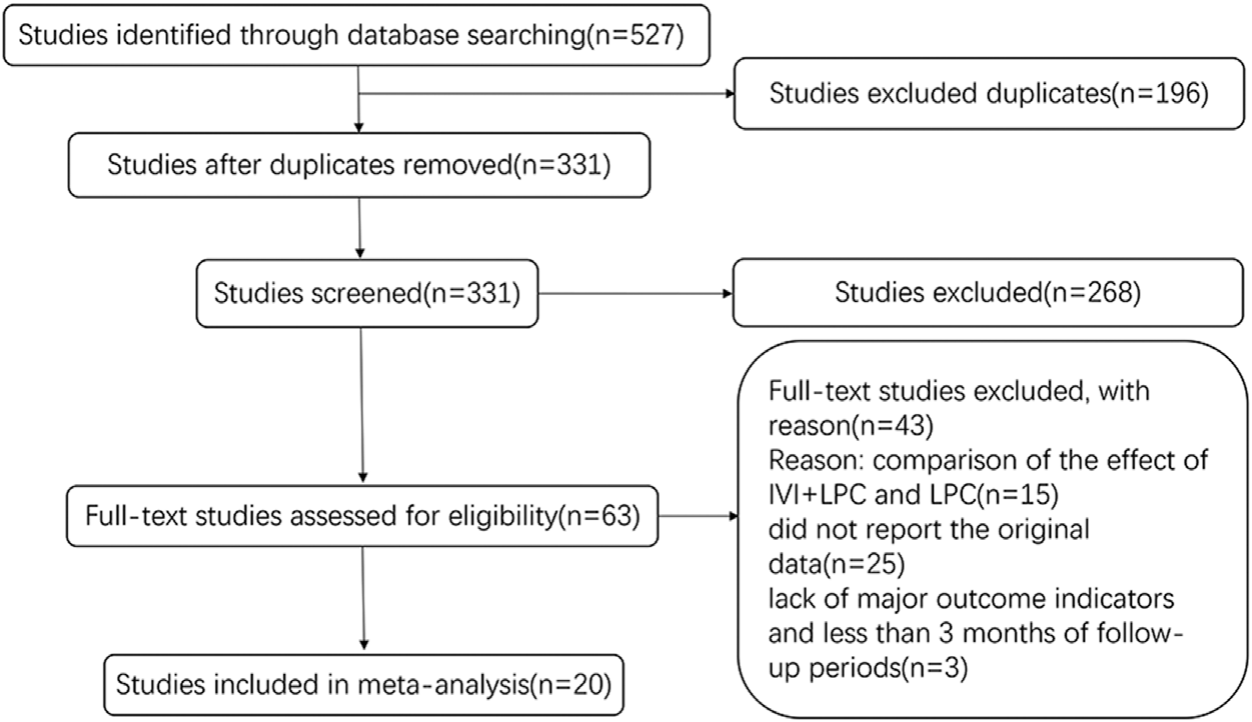
Flowchart of the study selection process.

A total of 527 studies were identified by database search from January 2011 to February 2022. 196 duplicates articles were excluded, and a total of 331 articles were retrieved. After the titles and abstracts were carefully reviewed, 268 irrelevant studies were removed. In the remaining 63 studies, 43 studies were excluded mainly because of the lack of original data. Finally, 20 studies were ultimately included in this meta-analysis study. The selection process is shown in Figure 1.

### Characteristics of included studies

A total of 1,387 patients with ME secondary to RVO were included in 20 studies including 16 randomized controlled trials and 4retrospective studies. The general data of all patients were shown in Table 1. There was no significant difference in age(t = -0.0954, *p* = 0.924) and sex(χ^2^ = 1.19, *p* > 0.05) between the IVI + LPC group and the single IVI group(Table 1).

**TABLE 1:** General imformation of the included trials.

Included studies	Location	Type of RVO	Interventions (Patients)	Age(years,‾x ± s)	Gender(M/F)	BCVA at baseline (letters,‾x ± s)	CMT at baseline (μm,‾x ± s)	Follow-up
Callizo et al. (2019)	Germany	BRVO	IVI + LPC(n = 16)	67.30 ± 9.30	10/6	0.55 ± 0.26 logMAR	496.10 ± 138.30	4,8,14,26,38 weeks
			IVI(n = 16)	67.50 ± 9.30	8/8	0.48 ± 0.25 logMAR	551.30 ± 160.40	
Cao et al. (2019)	China	BRVO	IVI + LPC(n = 20)	66.80 ± 5.09	11/9	0.85 ± 0.35 logMAR	620.30 ± 201.53	1,3,6,12 months
			IVI(n = 20)	69.80 ± 7.10	13/7	0.79 ± 0.27 logMAR	555.40 ± 115.57	
Chhablani et al. (2016)	India	CRVO	IVI + LPC(n = 11)	45.90 ± 8.10	NA	32.90 ± 14.99	870.00 ± 295.00	6–12 months
			IVI(n = 12)	52.46 ± 14.50	NA	39.20 ± 17.05	829.00 ± 332.00	
Goel et al. (2019)	India	BRVO	IVI + LPC(n = 16)	54.25 ± 9.56	6/10	0.88 ± 0.26 logMAR	496.69 ± 964.03	1,3,6,9 months
			IVI(n = 17)	55.88 ± 9.21	8/9	0.93 ± 0.26 logMAR	631.88 ± 964.03	
Tomomatsu et al. (2016)	Japan	BRVO	IVI + LPC(n = 19)	67.60 ± 8.32	9/10	NA	NA	1–6 months
			IVI(n = 19)	66.60 ± 9.06	7/12	NA	NA	
Kumar et al. (2019)	India	BRVO	IVI + LPC(n = 15)	57.93 ± 7.21	7/8	0.66 ± 0.14 logMAR	491.47 ± 92.01	1,3,6 months
			IVI(n = 15)	53.40 ± 5.32	8/7	0.68 ± 0.13 logMAR	487.53 ± 105.90	
Tadayoni et al. (2016)	America	BRVO	IVI + LPC(n = 180)	67.30 ± 10.41	96/84	56.60 ± 13.16	553.80 ± 170.06	1–6 months
			IVI(n = 183)	64.70 ± 10.34	93/90	59.50 ± 11.77	529.50 ± 144.97	
Hayashi et al. (2011)	Japan	BRVO	IVI + LPC(n = 19)	66.90 ± 12.00	NA	0.63 ± 0.40 logMAR	537.00 ± 174.00	4,8,12,24 weeks
			IVI(n = 25)	68.30 ± 11.00	NA	0.60 ± 0.41 logMAR	504.00 ± 197.00	
Donati et al. (2012)	Italy	BRVO	IVI + LPC(n = 9)	54.99 ± 1.58	NA	0.66 ± 0.24 logMAR	400.70 ± 49.10	1,2,3,6,12 months
			IVI(n = 9)	51.95 ± 1.40	NA	0.70 ± 0.25 logMAR	442.20 ± 98.60	
Farese et al. (2014)	Italy	BRVO	IVI + LPC(n = 19)	71.40 ± 8.00	11/8	0.50 ± 0.30 logMAR	475.30 ± 96.10	1,3,6,12 months
			IVI(n = 16)	69.80 ± 9.60	11/5	0.90 ± 1.10 logMAR	537.60 ± 181.20	
		CRVO	IVI + LPC(n = 10)	65.90 ± 11.00	5/5	0.70 ± 0.20 logMAR	630.20 ± 135.30	
			IVI(n = 9)	72.80 ± 15.40	3/6	0.90 ± 0.20 logMAR	568.40 ± 89.10	
Nourinia et al. (2020)	Iran	CRVO	IVI + LPC(n = 22)	NA	14/8	1.16 ± 0.62 logMAR	677.00 ± 229.00	1–9 months
			IVI(n = 24)	NA	17/7	1.06 ± 0.58 logMAR	675.00 ± 232.00	
An and Jeong, (2020)	Korea	BRVO	IVI + LPC(n = 17)	59.00 ± 8.49	5/12	0.60 ± 0.49 logMAR	NA	1–6 months
			IVI(n = 23)	61.52 ± 13.44	8/15	0.72 ± 0.43 logMAR	NA	
Terashima et al. (2019)	Japan	BRVO	IVI + LPC(n = 22)	67.90 ± 8.90	10/12	62.20 ± 12.00	515.00 ± 172.00	1–6 months
			IVI(n = 24)	68.80 ± 11.90	10/14	58.40 ± 21.30	513.00 ± 144.00	
Song et al. (2020)	China	BRVO	IVI + LPC(n = 34)	58.40 ± 9.70	18/16	54.40 ± 9.80	571.60 ± 223.50	1–12 months
			IVI(n = 30)	59.60 ± 11.00	17/13	59.30 ± 8.30	516.10 ± 161.10	
Rehak et al. (2014)	Germany	CRVO	IVI + LPC(n = 10)	63.70 ± 19.20	5/5	61.60 ± 12.70	452.60 ± 266.80	1–6 months
			IVI(n = 12)	63.70 ± 10.10	5/7	58.60 ± 12.50	466.70 ± 308.90	
Pielen et al. (2015)	Germany	BRVO	IVI + LPC(n = 10)	65.90 ± 11.20	6/4	0.41 ± 0.11 logMAR	505.60 ± 81.80	1,4,12,24 weeks
			IVI(n = 10)	64.20 ± 8.60	4/6	0.53 ± 0.24 logMAR	584.20 ± 250.90	
Clark et al. (2016)	America	BRVO	IVI + LPC(n = 90)	63.90 ± 11.40	54/36	57.70 ± 11.30	553.50 ± 188.10	1,6,12 months
			IVI(n = 91)	67.00 ± 10.40	44/47	58.60 ± 11.40	558.90 ± 185.90	
Thomley et al. (2021)	America	BRVO	IVI + LPC(n = 32)	73.50 ± 9.60	NA	NA	501.32 ± 223.04	1–36 months
			IVI(n = 56)	71.50 ± 10.00	NA	NA	484.12,140.73	
Ou et al. (2018)	America	RVO	IVI + LPC(n = 24)	64.10 ± 10.68	13/11	51.70 ± 20.20	525.50 ± 250.40	1,4,9,12 months
			IVI(n = 6)	63.80 ± 11.59	2/4	55.30 ± 21.70	471.50 ± 243.60	
Tultseva et al. (2017)	Russia	CRVO	IVI + LPC(n = 88)	62.50 ± 12.90	36/52	0.25 ± 0.15 logMAR	410.20 ± 157.25	1–28 months
			IVI(n = 87)	61.70 ± 11.40	34/53	0.27 ± 0.09 logMAR	425.50 ± 210.14	
BRVO, branch retinal vein occlusion. CRVO, central retinal vein occlusion. IVI, intravitreal injection. LPC, laser photocoagulation. BCVA, best corrected visual acuity. CMT, central macular thickness. NA, not available. Data are presented as mean ± standard deviation where applicable								

### Risk of bias assessment

**FIGURE 2:**
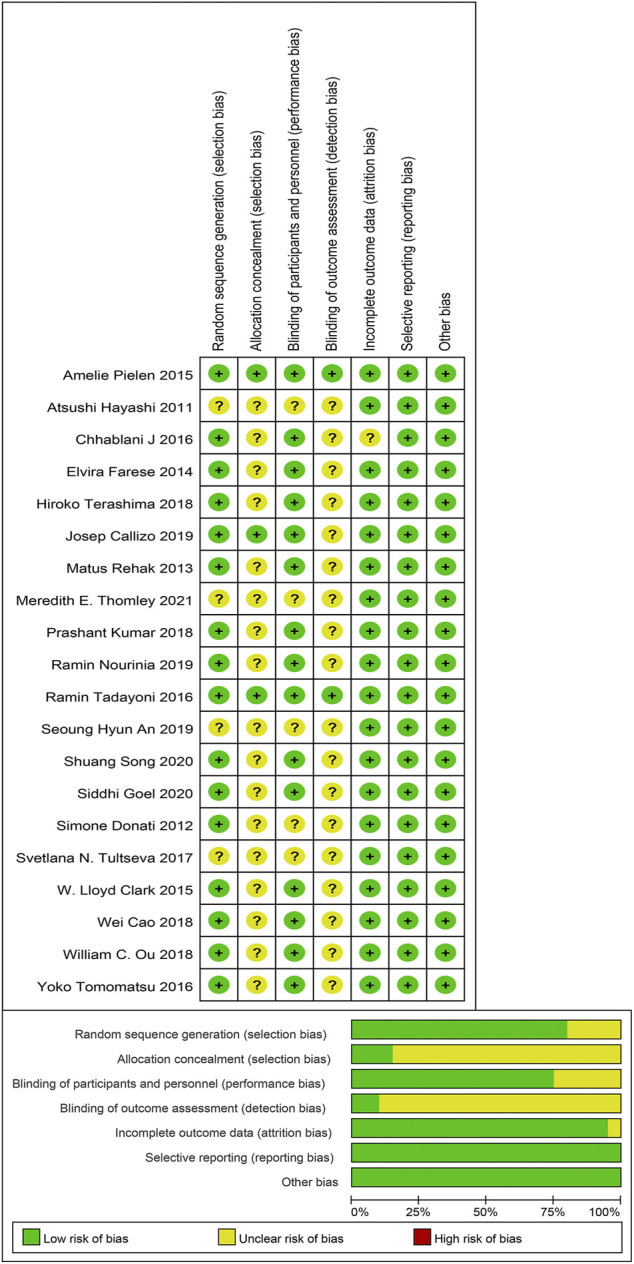
Risk of bias summary and Risk of bias graph.

Methodological quality and bias risk assessment showed that there was no high risk of bias in our included studies, while the unclear risk of bias was mainly focused on the allocation concealment and the blinding of outcome assessment. In a word, selection bias and detection bias were the most common biases in this study (Figure 2).

### Results of meta-analysis

**FIGURE 3:**
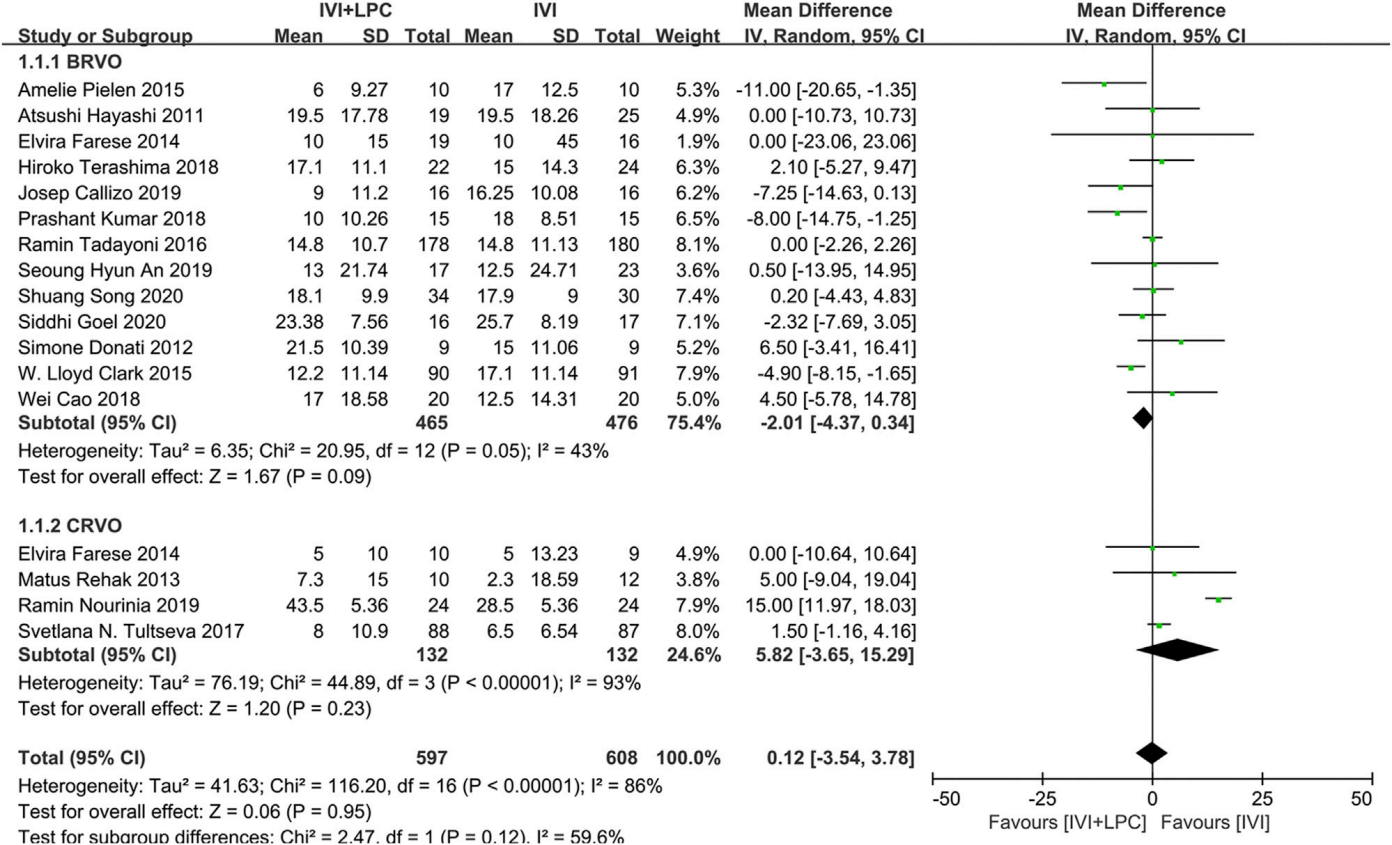
Comparison of BCVA between combination(IVI + LPC) treatment group and single IVI treatment group.

BCVA was reported in 16studies with a total of 1205 RVO patients before and after treatment, including 941 BRVO patients and 264 CRVO patients (Hayashi et al., 2011; Donati et al., 2012; Farese et al., 2014; Rehak et al., 2014; Pielen et al., 2015; Clark et al., 2016; Tadayoni et al., 2016; Tultseva et al., 2017; Callizo et al., 2019; Cao et al., 2019; Goel et al., 2019; Kumar et al., 2019; Terashima et al., 2019; An and Jeong, 2020; Nourinia et al., 2020; Song et al., 2020).The results of random effect model analysis(*p* < 0.00001,*I* ^*2*^ = 86%) showed that IVI is not inferior to IVI + LPC(*WMD* = 0.12,95%*CI =* -3.54–3.78,*p* = 0.95),neither in BRVO patients (*WMD* = -2.01,95%*CI =* -4.37–0.34,*p* = 0.09),nor in CRVO patients(*WMD* = 5.82,95%*CI = -*3.65–15.29,*p* = 0.23)(Figure 3).

**FIGURE 4:**
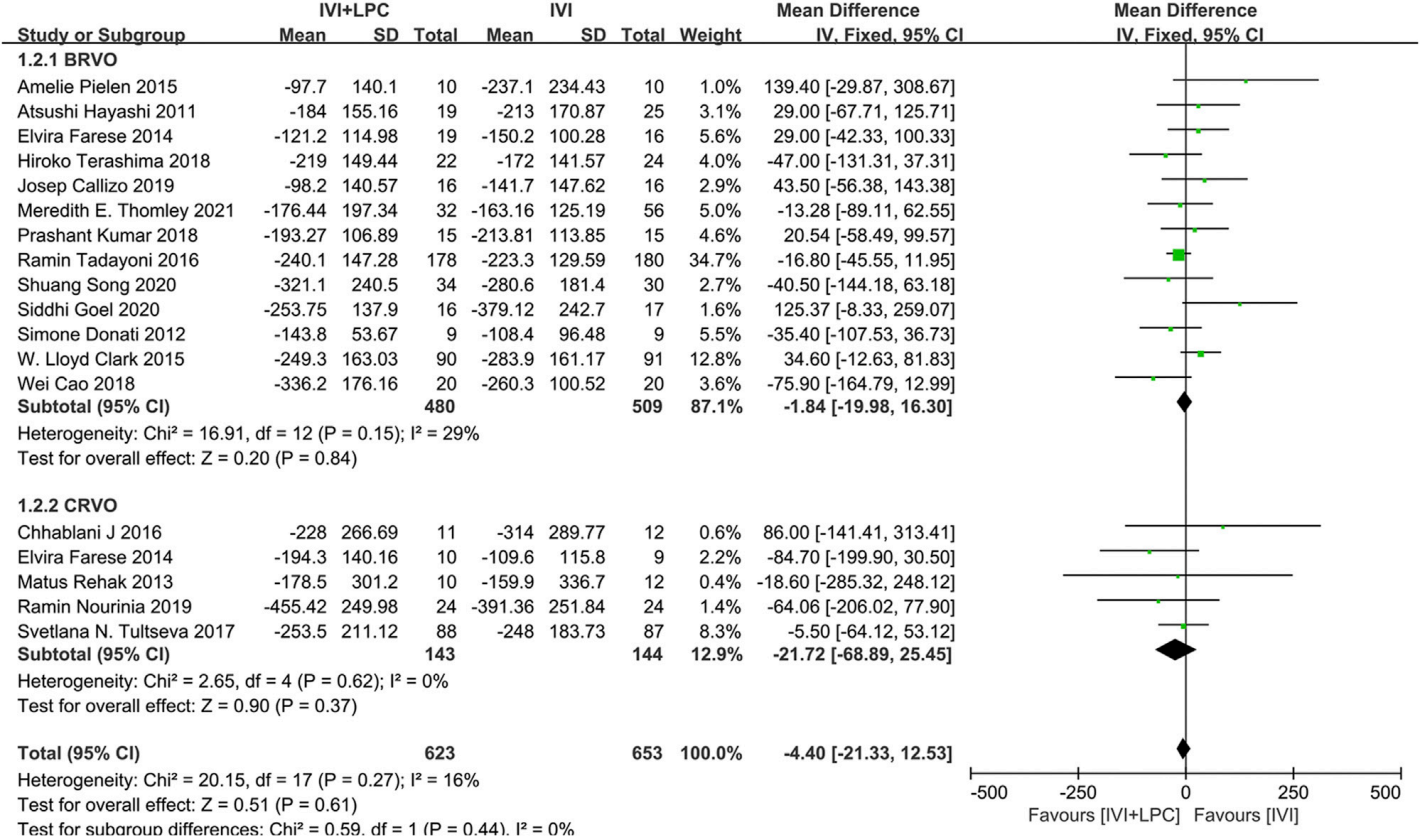
Comparison of CMT between combination(IVI + LPC) treatment group and single IVI treatment group.

The CMT was described in 17 studies with 1101 RVO patients including 989 BRVO patients and 287 CRVO patients(Hayashi et al., 2011; Donati et al., 2012; Farese et al., 2014; Rehak et al., 2014; Pielen et al., 2015; Chhablani et al., 2016; Clark et al., 2016; Tadayoni et al., 2016; Tultseva et al., 2017; Callizo et al., 2019; Cao et al., 2019; Goel et al., 2019; Kumar et al., 2019; Terashima et al., 2019; Nourinia et al., 2020; Song et al., 2020; Thomley et al., 2021).The fixed effect model analysis(*p* = 0.27,*I* ^*2*^ = 16%)demonstrated that there was no significant difference in CMT between IVI + LPC group and IVI group(*WMD* = -4.40,95%*CI =* -21.33–12.53,*p* = 0.61),whether in BRVO patients (*WMD* = -1.84,95%*CI =* -19.98–16.30,*p* = 0.84) or in CRVO patients(*WMD* = -21.72,95%*CI =* -68.89–25.45,*p* = 0.37)(Figure 4).

**FIGURE 5:**
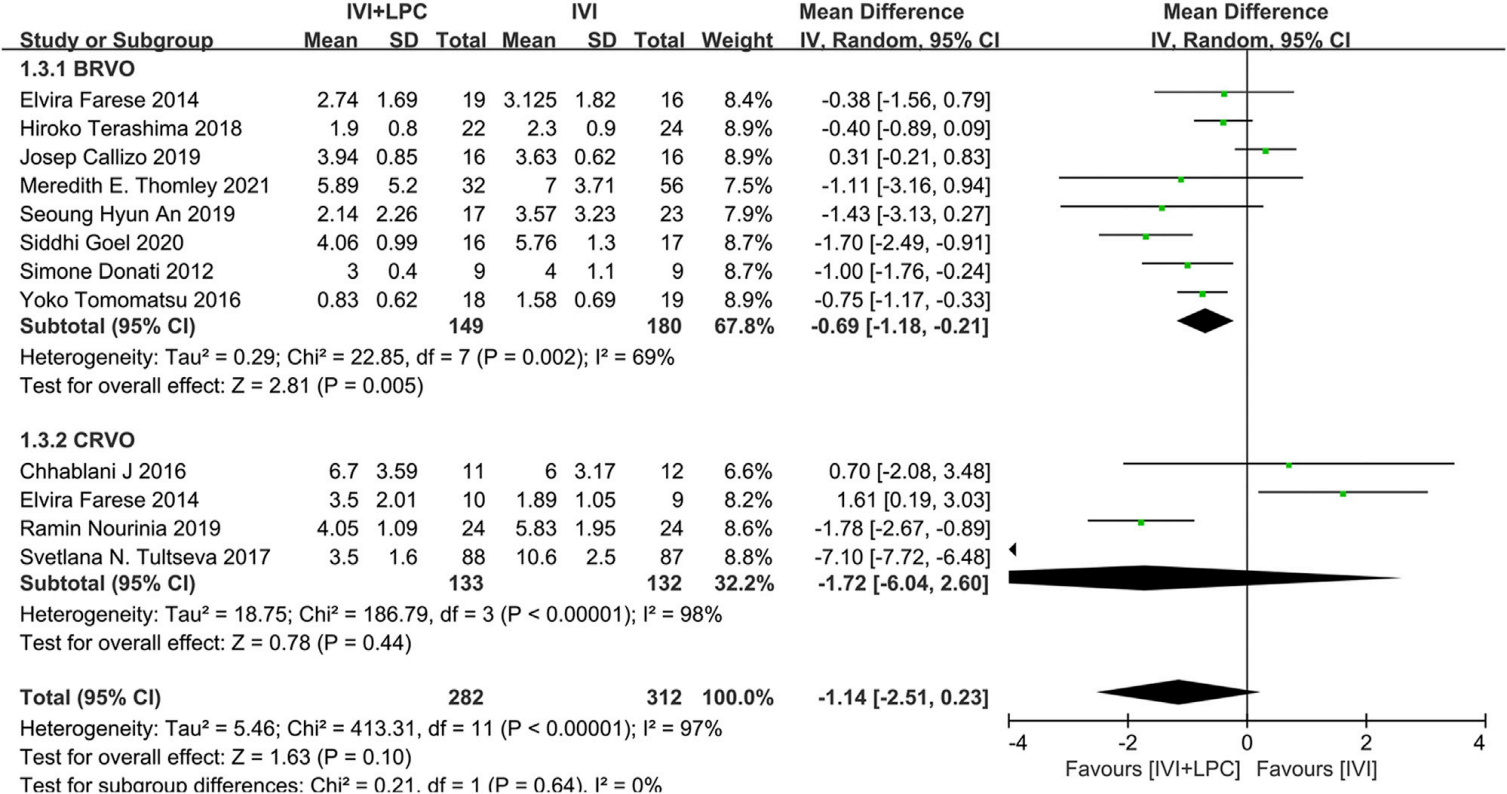
Comparison of the number of injections between combination(IVI + LPC) treatment group and single IVI treatment group.

The number of injections was recorded in 11 studies with 419 RVO patients including 329 BRVO patients and 265 CRVO patients(Donati et al., 2012; Farese et al., 2014; Chhablani et al., 2016; Tomomatsu et al., 2016; Tultseva et al., 2017; Callizo et al., 2019; Goel et al., 2019; Terashima et al., 2019; An and Jeong, 2020; Nourinia et al., 2020; Thomley et al., 2021).The results of the random effect model analysis(*p* < 0.00001, *I* ^*2*^ = 97%)illustrated that there was no significant difference between the IVI + LPC group and the single IVI group(*WMD* = -1.14,95%*CI =* -2.51–0.23, *p* = 0.10)(Figure 5).

Further subgroup analysis demonstrated that in BRVO patients with ME, the number of injections was sharply decreased in the combination treatment group compared with the single IVT treatment group (*WMD* = -0.69,95%*CI =* -1.18∼-0.21, *p* = 0.005). However, there was no significant difference in the number of injections between the combined treatment group and the single IVI group in CRVO patients with ME (*WMD* = -1.72,95%*CI =* -6.04–2.60, *p* = 0.44)(Figure 5).

**FIGURE 6:**
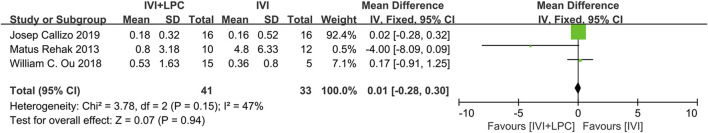
Comparison of the change of NPAs areas between combination(IVI + LPC) treatment group and single IVI treatment group.

The progress of NPAs was recorded and quantified by three studies with 74 RVO patients(Rehak et al., 2014; Ou et al., 2018; Callizo et al., 2019). The results of the fixed effect model analysis(*p* = 0.15, *I* ^*2*^ = 47%) showed that no significant differences were detected in the change of NPAs areas between the combined treatment group and the single IVI group (*WMD* = 0.01,95%*CI =* -0.28–0.30, *p* = 0.94)(Figure 6).

## Discussion

At present, the main treatments for RVO patients with ME include intravitreal injection of anti-VEGF or corticosteroid drugs, retinal laser photocoagulation and surgeries. The IVI of anti-VEGF drug therapy has been recommended as the first-line clinical medication, because of its outstanding effect in improving the visual acuity and reducing ME. However, the medical risks (such as infection and cardiovascular accidents) and the economic burden should not be ignored, as it needs repeated injections. Therefore, how to decline the number of IVI treatment in RVO patients is one of the current research hotspots. Recently, some studies(Donati et al., 2012; Farese et al., 2014; Rehak et al., 2014; Tomomatsu et al., 2016; Tultseva et al., 2017; Goel et al., 2019; Terashima et al., 2019; An and Jeong, 2020; Nourinia et al., 2020) have found that, compared with single IVI, the combination of IVI and LPC could improve the prognosis in RVO patients with ME and decrease the number of injections. However, other studies(Hayashi et al., 2011; Pielen et al., 2015; Chhablani et al., 2016; Tadayoni et al., 2016; Callizo et al., 2019; Cao et al., 2019; Kumar et al., 2019; Song et al., 2020; Thomley et al., 2021) suggested that combination treatment could neither improve the prognosis of patients nor reduce the frequency of IVI, and on the contrary, it has increased the medical burden of RVO patients.

The results of this study demonstrated that both the combined treatment and single IVI treatment can effectively improve BCVA and decrease CMT in RVO patients with ME. However, no obvious evidence showed that additional laser photocoagulation could further amplify the benefits of IVI on BCVA and CMT. In addition, the combined treatment can reduce the number of injections conspicuously in BRVO patients with ME, however, the difference was not found in CRVO patients. The results suggested that additional laser photocoagulation cannot delay the progress of NPAs in CRVO patients with ME, while it may be helpful to the progress of NPAs in BRVO patients.

A possible mechanism is that combined (IVI + LPC) treatment may slow down the progress of retinal non-perfused areas (NPAs) in BRVO patients. Previous studies(Stefánsson, 2001; Tomomatsu et al., 2016) have proved that hypoxia plays an important role in the pathogenesis of RVO with ME. Hypoxia increases the expression of VEGF and vascular permeability, which leads to vascular leakage and macular edema. Anti-VEGF drugs can effectively inhibit this process and decline the severity of ME by reducing the damage to the blood-retinal barrier. However, because of the short half-life of anti-VEGF drugs, the intraocular drug concentration decreases rapidly, and the peripheral retinal NPAs continue to release VEGF, resulting in the recurrence of ME. Some studies(Rehak et al., 2014; Tan et al., 2016) have shown that the size of NPAs is related to the severity of BRVO, and after being combined with LPC, it partially destroyed the peripheral retinal pigment epithelia and photoreceptors, declined the tissue hypoxia and inhibit the release of VEGF from retinal NPAs. And eventually, it slowed down the progress of NPAS.

Some studies(Kokolaki et al., 2015; An and Jeong, 2020)also suggested that early LPC treatment could promote the formation of collateral vessels in retinal NPAs in BRVO patients. With the extension of time after laser photocoagulation, the number of collateral vessels parallelly increases at the same time. Finally, the time of collateral vessels formation in patients with combined therapy (IVI + LPC) is less than that in patients with anti-VEGF drugs alone(Rehak et al., 2014). By declining NPAs, additional LPC could improve the function of the collateral vessels and inhibit the release of VEGF. Then, it ameliorates the state of hypoxia and oxidative stress in the whole retina and delays the recurrence of macular edema.

On the other hand, for patients with CRVO, it is not clear why combined treatment cannot improve the prognosis or reduce the number of IVI. The possible reasons are as follows: (1) **As it is known that, CRVO can be divided into ischemic CRVO and nonischemic CRVO, ischemic CRVO has a risk of neovascularization(NV) while there is no risk of NV associated with nonischemic CRVO. Some researchers thought that laser photocoagulation could play an important role in the treatment of NV, thus improving the prognosis of ischemic CRVO patients. However, for nonischemic CRVO patients, LPC could have no treatment effect (** **Hayreh, 2021** **). We could not conduct a more detailed analysis because of the limitation of the data we extracted in our included studies. Therefore, our results may be influenced by the absence of hierarchical analysis of CRVO.** (2) Compared to the relatively limited pathological changes in BRVO, the larger retinal areas were affected by CRVO, which may diminish the efficiency of additional LPC. (3)Althoughpan-retinal photocoagulation (PRP) for CRVO patients could reduce the level of intraocular VEGF, it also causes the incline of intraocular inflammatory factors, which is not beneficial for the regression of macular edema. (4) Pan-retinal photocoagulation can close the capillary NPAs, however, it still leads to a wide range of non-vascularized areas around NPAs in CRVO. And the perfusion of the whole retina cannot match the demand for blood oxygen of the retina, as the intraocular VEGF is continuously released. Thus the combination of IVI and retinal LPC cannot delay the recurrence of CRVO with macular edema.

To our knowledge, this meta-analysis is the first study that concludes all the available research data in the recent 10 years of intravitreal injection of anti-VEGF drugs and retinal laser photocoagulation in RVO patients with ME. However, the limitations of this study are as follows: (1)The published clinical research data is still not sufficient because of the lack of indicators for the treatment of RVO in some anti-VEGF drugs. (2)Because the related studies involving different types of anti-VEGF drugs, varies greatly, the heterogeneity and publication bias of statistical analysis cannot be avoided. (3)**Considering the limitation of the data we extracted in our included studies, some detailed analysis cannot be conducted between subgroups, which may influence our final results.** Therefore, it is necessary to include more studies for further analysis in the future.

## Data Availability

The original contributions presented in the study are included in the article/Supplementary Material, further inquiries can be directed to the corresponding author.
